# Burnout Syndrome Amongst Medicine Students in Lithuania and Germany

**DOI:** 10.15388/Amed.2020.27.2.2

**Published:** 2020-12-21

**Authors:** Ieva Rudinskaitė, Eglė Mačiūtė, Giedrė Gudžiūnaitė, Greta Gerulaitytė

**Affiliations:** Vilnius University, Faculty of Medicine, Lithuania; Vilnius University, Faculty of Medicine, Lithuania; Vilnius University, Faculty of Medicine, Lithuania; Vilnius University, Faculty of Medicine, Lithuania

**Keywords:** burnout, medical students, stress

## Abstract

**Summary. Background.:**

The research provides comparative analysis of the burnout syndrome among medical students in Lithuania and Germany and determines relations between burnout and lifestyle, health complaints and seeking for psychological help.

**Materials and methods.:**

The research was conducted in April 2019 using an anonymous self-administered e-based questionnaire. The sample size was 261 medical students (age mean 20,5 ± 1,8, 46 males, 215 females): 131 from Vilnius University (VU), 67 from Lithuanian University of Health Science (LSMU) and 63 from Heidelberg University (HU), Germany. The 15 items MBI-SS scale was used to identify burnout syndrome among students *(Cronbach α =0,74).* Emotional exhaustion (>14), cynicism (>6), and professional efficacy (<23) scores were calculated for academic burnout. The participants were also asked additional questions related to lifestyle and well-being. Descriptive statistics were analysed using MS Office Excel, SPSS and R Commander, results were statistically significant when *p<0,05.*

**Results.:**

51 (38,9%) VU, 25 (37,3%) LSMU and 10 (15,9%) HU students were ascertained as burnt-out *(p=0,004)*. 76 (90,6%) respondents complained of feeling study induced mental stress *(p=0,0002)*. Furthermore, 67 (77,9%) respondents indicated having a bad mood *(p=0,043)*, 54 (62,7%) general weakness* (p=0,024)*, 44 (51,1%) digestive problems* (p=0,003)*. Sleep duration was strongly associated with burnout *(p=0,002)* with over half (n=51, 59,3%) of the burnout respondents sleeping <7 hours a day. 87 (33,3%) students considered consulting a psychologist, but only 24 (9,2%) reached out for help.

**Conclusions.:**

More than 1/3 of medical students in the research group had signs of burnout. The highest prevalence of burnout was among VU and lowest among HU students. Burnt-out respondents complained of mental stress, weakness, bad mood, digestive problems. The university studies and the duration of sleep can impact the occurrence of burnout syndrome. Only every forth student, willing to consult a psychologist, sought assistance.

## Abstract (lt)

**Lietuvos ir Vokietijos medicinos studentų perdegimo sindromas:**

**Santrauka.**

**Įvadas.**

Tai Lietuvos ir Vokietijos medicinos studentų perdegimo tyrimas, kuriame pateikiama lyginamoji analizė, nagrinėjamos perdegimo sindromo ir gyvenimo būdo, nusiskundimų sveikata ir psichologinės pagalbos sąsajos.

**Metodika.**

Tyrimas atliktas 2019 metų balandžio mėn. taikant anoniminės anketinės apklausos internete metodą. Tiriamųjų grupę sudarė 261 (amžiaus vidurkis 20,5 ± 1,8, 46 vyrai, 215 moterų) tiriamasis – I–VI kurso medicinos studentai iš Lietuvos ir Vokietijos universitetų: 131 Vilniaus universiteto (VU), 67 Lietuvos sveikatos mokslų universiteto (LSMU) ir 63 Heidelbergo universiteto (HU) medicinos studentai. Perdegimo sindromui nustatyti buvo panaudota speciali 15 teiginių MBI-SS skalė (studentams adaptuota Maslach perdegimo skalė, angl. – *Maslach Burnout Inventory-Student Survey*) (Cronbach α=0,74). Akademiniam perdegimui įvertinti buvo apskaičiuoti emocinio išsekimo (>14), cinizmo (>6) bei profesinio veiksmingumo (<23) balai. Taip pat dalyviams buvo užduota papildomų klausimų, susijusių su gyvensena ir savijauta. Surinkti duomenys apdoroti MS Office Excel, SPSS bei statistine R Commander programa. Rezultatų skirtumas laikytas statistiškai reikšmingu, kai p reikšmė <0,05.

**Rezultatai.**

51 (38,9%) VU, 25 (37,3%) LSMU ir 10 (15,9%) HU studentų buvo nustatytas akademinis perdegimas įvertinus emocinio išsekimo, cinizmo bei profesinio veiksmingumo balus *(p=0,004)*. Net 76 (90,6%) perdegimą patyrę respondentai skundėsi dėl studijų išgyvenama nervine įtampa *(p=0,0002)*, 67 (77,9%) bloga nuotaika *(p=0,043)*, 54 (62,7%) bendru silpnumu *(p=0,024)*, 44 (51,1%) – virškinimo sutrikimais *(p=0,003)*. Nustatytas statistiškai reikšmingas perdegimo ir miego trukmės ryšys *(p=0,002)*. Daugiau nei pusė (n=51, 59,3%) respondentų, kuriems būdingas akademinis perdegimas, miega mažiau nei septynias valandas. 87 (33,3%) tyrime tyrime dalyvavę studentai būtų linkę pasikonsultuoti su psichologu, tačiau tik 24 (9,2%) kreipėsi pagalbos.

**Išvados.**

Akademinis perdegimas nustatytas daugiau nei trečdaliui tyrimo respondentų. Akademinis perdegimas plačiausiai paplitęs tarp VU, mažiausiai – tarp Vokietijos HU medicinos fakultetų studentų. Dauguma respondentų, kuriems nustatytas akademinis perdegimas, skudėsi patiriantys nervinę įtampą, bendrą silpnumą, blogą nuotaiką, virškinimo sutrikimus. Galima daryti prielaidą, kad universitetas ir miego trukmė lemia akademinio perdegimo atsiradimą. Maždaug tik kas ketvirtas studentas, kuris būtų linkęs konsultuotis su psichologu, kreipiasi pagalbos.

**Raktažodžiai:** perdegimas, medicinos studentai, stresas

## Introduction

In our busy day and age, the number of people that suffer from stress, anxiety and negative emotions is increasing every single day [1-3]. All of these reasons can encourage the manifestation of burnout [4,5]. Burnt-out people start to feel incompetent, sad and guilty. They lose their motivation and purpose. Burnout has a negative effect not only to a person suffering from it but also towards his/her relationships. This dreary state can initiate the end of relationships, the rise of depressive or suicidal thoughts. A study conducted in Poland discovered that people who are not prone to “burning-out” or are burnt-out at a minor level, are more content with their relationships, health and work. On the other hand, people experiencing medium to high levels of burnout are usually dissatisfied with their financial situation, complain of the lack of free time and troubled relationships with their friends and family. In other words, burnout syndrome, in one way or another, impacts the quality of person’s life [6].

Maslach and Jackson described burnout as a syndrome composed of three dimensions: deper-sonalization (cynicism), emotional exhaustion, and a lack of personal accomplishment [7]. C. Ma-slach’s – one of the most notorious researchers of this syndrome – research revealed that during burnout one of the first symptoms is the loss of emotions and senses, and only later is it followed by a negative outlook towards one’s self and others. In that sense, burnout is regarded not as a disease, but as an organic whole of symptoms (a syndrome).

Academic burnout is a far newer (2002) and more rarely discovered topic in literature in comparison to the earlier described professional burnout [8]. It is worth noting that academic burnout is becoming a common problem among university and college students alike. The indications of academic burnout include emotional drain, cynicism and a decrease in learning productivity. Academic burnout varies from professional as only the well-being related towards the study process is considered, whilst relationships are excluded. Medical students are often selected as study subjects of the matter as due to the vast amount of information, high standards and limited free time, they are among the most prone to lose connection with themselves and to burnout [9]. Studies show that even 33% medical students abuse alcoholic substances, whereas only 16% of nonmedical students in the same age group face this problem [10]. Burnt-out students experience the following psychological and clinical symptoms: depression, insomnia, anxiety, inability to concentrate, headaches and digestion issues [11].

## Material and methods

### Study design and population

This was an observational cross-sectional study which was carried out at three medical schools: Vil-nius University (VU), Lithuanian University of Health Sciences (LSMU) and Heidelberg University (HU). The study was conducted in April 2019 and involved 261 medical students.

### Eligibility criteria

Students included in the study were those enrolled in the six years undergraduate medical course who voluntarily agreed to answer the questionnaire.

### Instrument

Medical students were asked to complete a self-administered e-based questionnaire. The academic burnout was assessed using MBI-SS (*Maslach Burnout Inventory-Student Survey*)*. *WB. Schaufeli, M. Salanova, BA. Bakker in 2002 had adapted MBI-GS to Spanish, Portuguese and Dutch students [8]. The MBI-SS consists of 15 statements divided into three subscales representing academic burnout: emotional exhaustion (five statements), cynicism (four statements), and professional efficiency (six statements). Responses on the MBI-SS range on a six-point Likert-scale from never to always. The three dimensions (high scores for emotional exhaustion and cynicism and low scores for academic efficiency) were used as the criteria for the diagnosis of academic burnout. The alpha Cron-bach statistic for emotional exhaustion items was 0,9 while that for cynicism items was 0,91, and for professional efficiency items was 0,84. When three subscales were combined, the Cronbach’s alpha value for this study was 0,74. The sociodemographic characteristics also were assessed: gender, age, marital status, leisure time, physical activity, sleep duration, eating habits, smoking, drinking, health issues. In order to evaluate academic work and regime students were asked about the attendance of lectures and time spent for self-study. 

### Data analysis

Various statistical variables were evaluated: sample size, median, mean, standard deviation and frequency. The Kolmogorov–Smirnov test was used to test the normality of quantitative variables, as the sample contains 261 medical students. For qualitative variables the frequencies and percentages were calculated. Categorical variables were compared using Chi-square (χ2) and Fisher exact tests, and qualitative variables were compared using Wilcoxon test. The MS Office Excel, SPSS statistics and R Commander were used for the analysis. Differences were considered as statistically significant when p< 0,05.

## Results

Two hundred sixty-one medical students from VU, LSMU and HU participated in this study. The age mean of the research group is 20,5 ± 1,8, of which women made up 215 (82,4%), men – 46 (17,6%). 51 (38,9%) VU, 25 (37,3%) LSMU and 10 (15,9%) HU students were ascertained as burnt-out aﬅer evaluating emotional exhaustion, cynicism and professional efficacy scale and the acquired difference was statistically significant (*p=0,004*). 

*The Table*shows sociodemographic characteristics among students with burnout. The largest prevalence of burnout syndrome was found among course IV (n=12, 50%), V (n=3, 33,3%) and I (n=40, 31,4%) medical students. The majority of medicine students with burnout are single (n=57, 37,01%) or in a relationship (n=21, 25,3%). Sleep duration was strongly associated with the burnout (*p=0,002*)**with over half (59,3%, n=51) of the burnout respondents sleeping <7 hours a day. 

Also, more than a third (n=100, 38,3%) of the respondents do not know what kind of health status is determined by the term “burn-out syndrome”.

**Figure 1.**
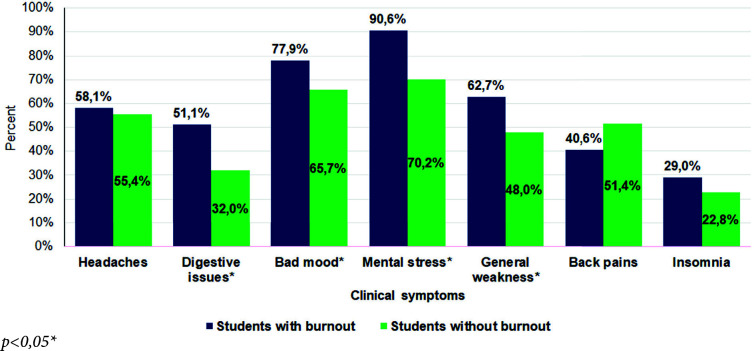
Clinical symptoms between student groups with burnout and without burnout.

*The Figure 1*shows clinical symptoms between students with burnout and without burnout. 78 (90,6%) respondents with burnout suffer from mental stress, 67 (77,9%) bad mood (*p=0,043*), 54 (62,7%) general weakness**(*p=0,024*), 44 (51,1%) digestive problems**(*p=0,003*). On the other hand, the majority of respondents without burnout suffer from mental stress (70,2%), bad mood (65,7%) and headaches (55,4%). The rarest symptom in both groups was insomnia.

**Figure 2.**
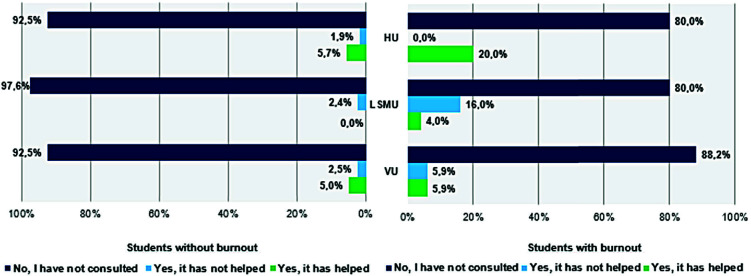
Necessity of a psychologist help between student groups with and without burnout in different universities.

*In Figure 2* the necessity of a psychologist help between student groups with and without burnout is shown. It can be seen that the majority of respondents have not consulted with a psychologist. Students with burnout are more likely to get psychological help. 87 (33, 3%) of the medical students who participated in the study would be willing to consult a psychologist, but only 24 (9,2%) reached out for help.

**Table.** Sociodemographic characteristics among students with burnout.

Variables	With Burnout N (%)			p
	VU	LSMU	HU	
***Academic burnout ***				0,004*
	51 (38,9)	25 (37,3)	10 (15,9)	
***Gender***				0,957
Female	44 (86,3)	20 (80)	7 (10)
Male	7 (13,7)	5 (20)	3 (3)	
***Undergraduate school year***				0,367
I	20(39,2)	16 (64)	4 (40)	
II	16(31,4)	4 (16)	3 (30)	
III	5 (9.8)	1 (4)	1 (10)	
IV	10 (19,6)	2 (8)	0 (0)	
V	0 (0)	2 (8)	1 (10)	
VI	0 (0)	0 (0)	1 (10)	
***Work***				0,054
Yes	4(7,8)	4(16)	3(3)	
No	47(92,2)	21(84)	7(7)	
***Marital status***				0,459
Single	35(68,5)	17(68)	5(50)	
Married	1(2)	0(0)	0(0)	
Living together but not married	2(4)	1(4)	3(30)	
In a relationship	13(25,5)	6(24)	2(20)	
Divorced/separated	0(0)	1(4)	0(0)	
***Independent studying***				0,883
1-5 val.	34(66,7)	17(68)	6(60)	
6-10 val.	14(27,4)	6(24)	4(40)	
11-15 val.	3(5,9)	1(4)	0(0)	
16-20 val.	0(0)	1(4)	0(0)	
***Leisure time***				0,493
< 1 val.	9(17,7)	1(4)	2(20)	
1-2 val.	23(45,1)	8(32)	4(40)	
3-4 val.	15(29,4)	10(40)	2(20)	
> 4 val.	4(7,8)	6(24)	2(20)	
***Sleep time***				0,002*
3-4 val.	2(4)	2 (8)	0(0)	
5-6 val.	27(52,9)	15(60)	5 (50)	
7-8 val.	21(41,1)	8(32)	5 (50)	
9-10 val.	1(2)	0(0)	0(0)	
***Lecture attendance***				0,361
0-25%	2(4)	1(4)	2(20)	
26-50%	7(13,7)	2(8)	2(20)	
51-75%	11(21,6)	5(20)	1(10)	
76-100%	31(60,7)	17(68)	5(50)	
***Meals per day***				0,572
Once	3(5,9)	1(4)	1(10)	
Twice, three times	38(74,5)	16(64)	9(90)	
Four or more times	10(19,6)	8(32)	0(0)	
***Smoking***				0,718
Daily	2(4)	4(16)	0(0)	
Sometimes	11(21,5)	5(20)	1(10)	
Not at all	38(74,5)	16(64)	9(90)	
***Consumption of alcohol***				0,269
Everyday	0(0)	0(0)	0(0)	
2-3 times a week	2(4)	1(4)	1(10)	
Once a week	4(7,8)	2(8)	4(40)	
2-3 times a month	21(41,1)	8(32)	1(10)	
A few times a year	16(31,4)	7(28)	3(30)	
I do not drink alcohol beverages	8(15,7)	7(28)	1(10)	

## Discussion

The aim of the presented study was to investigate the prevalence of burnout syndrome among Lithuanian and German medical students by assessing their lifestyle and other sociodemographic characteristics. Th e study showed that burnout syndrome has a significant impact on well-being and mental health of the surveyed students. Th ere are many causes of burnout and each additional stressor increases the risk of more serious consequences for mental health, but there is also a significant cause-and-effect difference between developed and developing countries. In terms of developed countries, in Lithuania and Germany we have noticed reduced sleep time, alcohol abuse and irregular meals during the day. Medical studies are heavily burdened with psychological stress factors, and the addition of conflicts between family and career could lead students to a higher risk of being burnt out. According to another study, if medical students were encouraged to adopt healthier lifestyle changes in their daily lives from the very beginning of their studies, burnout outbreaks and their following consequences could be prevented or at least reduced in their further careers [12]. 

It should be noted, that it is significant not only to understand the causes and consequences of burnout, but also ways to reduce the risk of experiencing psychological distress or mitigate the already existing negative psychological consequences. 

Nowadays, burnout syndrome is increasingly being recognised as a significant mental health issue in global health around the world. However, like several mental health disorders, this syndrome is highly neglected. Most interviewees with established burnout syndrome do not seek psychological help or claim that psychologist services were ineffective, especially at Lithuanian universities. Although some students clearly understand the need and benefits of psychological help, there are some barriers that discourage young people from seeking professional psychologist’s or psychotherapist’s advice. According to another study, the most common barrier to seeking psychotherapy seems to be public and self-stigma. Some decide to stop consulting psychologists aﬅer a first visit they believe to be unsuccessful, because they no longer believe in the success of the process. Yet other medical students choose not to seek for specialists because of financial costs, especially when the sources of income are extremely limited and studying takes up most of their time [12]. So even if medical students are usually aware of the advantages of psychotherapy for their patients, they oﬅen do not accept themselves as people who also need professional help.

## Conclusions

The results of the study confirm that more than a third of medical students in the research group had signs of burnout. The highest prevalence of the burnout was among VU and the lowest one among HU students. Burnt-out respondents complained of mental stress, weakness, bad mood, digestive problems. The study also revealed that the university studies and duration of sleep can impact the occurrence of burnout syndrome. Only every forth student, willing to consult a psychologist, sought assistance.

## References

[ref1] Koutsimani P, Montgomery A, Georganta K. The Relationship Between Burnout, Depression, and Anxiety: A Systematic Review and Meta-Analysis. *Front Psychol*. 2019;10:284. Published 2019 3 13. doi:10.3389/fpsyg.2019.00284 30918490 10.3389/fpsyg.2019.00284 PMC6424886

[ref2] Minter, R. L. (2009). Faculty Burnout. *Contemporary Issues in Education Research (CIER) 2009;* 2(2):1-8. 10.19030/cier.v2i2.1090

[ref3] Shadman N, Raoof M, Amanpour S, Mahdian M, Haghani J, Torabi Parizi M. Stress, Anxiety, and Depression and Their Related Factors Among Dental Students: A Cross-Sectional Study from Southeast of Iran. *Strides in Development of Medical Education*. 2019; 6(1):e74295. doi: 10.5812/sdme.74295

[ref4] Lyndon MP, Henning MA, Alyami H, et al. Burnout, quality of life, motivation, and academic achievement among medical students: A person-oriented approach. *Perspect Med Educ*. 2017;6(2):108-114. doi:10.1007/s40037-017-0340-6 28247209 10.1007/s40037-017-0340-6 PMC5383573

[ref5] Riethof N, Bob P. Burnout Syndrome and Logotherapy: Logotherapy as Useful Conceptual Framework for Explanation and Prevention of Burnout. *Front Psychiatry*. 2019;10:382. Published 2019 6 14. doi:10.3389/fpsyt.2019.00382 31258490 10.3389/fpsyt.2019.00382 PMC6587911

[ref6] Śliwiński Z, Starczyńska M, Kotela I, Kowalski T, Kryś-Noszczyk K, Lietz-Kijak D, ir kt. Life satisfaction and risk of burnout among men and women working as physiotherapists. *Int J Occup Med Environ Health*. 2014; 27(3):400–12. 10.2478/s13382-014-0266-8 24820030

[ref7] Kaplan JA, Weichenthal L. Wellness, Stress, and the Impaired Physician. In: *Rosen’s Emergency Medicine: Concepts and Clinical Practice*. 9th ed. Philadelphia, PA: Elsevier; 2018. https://www.clinicalkey.com/#!/content/book/3-s2.0B9780323354790002208?scrollTo=%23hl0000089

[ref8] Schaufeli WB, Martníez IM, Pinto AM, Salanova M, Bakker AB. Burnout and Engagement in University Students: A Cross-National Study. *J Cross-Cult Psychol*. 2002; 33(5):464–81.

[ref9] Cecil J, McHale C, Hart J, Laidlaw A. Behaviour and burnout in medical students. *Medical Education Online*. 2014; 19:1, doi: 10.3402/meo.v19.25209 10.3402/meo.v19.25209 PMC4145104 25160716

[ref10] Jackson ER, Shanafelt TD, Hasan O, Satele DV, Dyrbye LN. Burnout and Alcohol Abuse/Dependence Among U.S. Medical Students. Acad Med. 2016 9;91(9):1251-6. doi: 10.1097/ACM.0000000000001138. 26934693 10.1097/ACM.0000000000001138

[ref11] Burnout: Causes, Symptoms & Diagnostics - Schoen Clinic. https://www.schoen-clinic.com/burnout [accessed 3 5, 2020]

[ref12] Constantinou CS, Georgiou M, Perdikogianni M. Medical Students’ Attitudes and Beliefs towards Psychotherapy: A Mixed Research Methods Study. *Behav Sci (Basel)*. 2017;7(3):55. Published 2017 8 18. doi:10.3390/bs7030055 10.3390/bs7030055 PMC5618063 28820440

